# Sustained Release of Levobupivacaine, Lidocaine, and Acemetacin from Electrosprayed Microparticles: In Vitro and In Vivo Studies

**DOI:** 10.3390/ijms21031093

**Published:** 2020-02-06

**Authors:** Jian-Ming Chen, Kuan-Chieh Liu, Wen-Ling Yeh, Jin-Chung Chen, Shih-Jung Liu

**Affiliations:** 1 Department of Orthopedic Surgery, Chang Gung Memorial Hospital-Linkou, Tao-Yuan 33305, Taiwan; 2 Department of Mechanical Engineering, Chang Gung University, Tao-Yuan 33302, Taiwan; 3 Graduate Institute of Biomedical Science, Chang Gung University, Tao-Yuan 33302, Taiwan; Jinchen@mail.cgu.edu.tw

**Keywords:** sustained drug release, poly(D,L-lactide-co-glycolide), electrospraying, acemetacin, levobupivacaine, lidocaine

## Abstract

In this study, we explored the release characteristics of analgesics, namely levobupivacaine, lidocaine, and acemetacin, from electrosprayed poly(D,L-lactide-co-glycolide) (PLGA) microparticles. The drug-loaded particles were prepared using electrospraying techniques and evaluated for their morphology, drug release kinetics, and pain relief activity. The morphology of the produced microparticles elucidated by scanning electron microscopy revealed that the optimal parameters for electrospraying were 9 kV, 1 mL/h, and 10 cm for voltage, flow rate, and travel distance, respectively. Fourier-transform infrared spectrometry indicated that the analgesics had been successfully incorporated into the PLGA microparticles. The analgesic-loaded microparticles possessed low toxicity against human fibroblasts and were able to sustainably elute levobupivacaine, lidocaine, and acemetacin in vitro. Furthermore, electrosprayed microparticles were found to release high levels of lidocaine and acemetacin (well over the minimum therapeutic concentrations) and levobupivacaine at the fracture site of rats for more than 28 days and 12 days, respectively. Analgesic-loaded microparticles demonstrated their effectiveness and sustained performance for pain relief in fracture injuries.

## 1. Introduction

Fractures are common injuries with a prevalence rate of approximately six in a thousand and are usually the result of an intense mechanical stress to the body [1]. Furthermore, bone fractures can also result from certain medical conditions that weaken the bones, such as bone cancer or osteoporosis, which make even low-impact traumas harmful. Both the fracture itself and any injuries to the surrounding soft tissue lead to severe pain. Simple fractures can be treated conservatively by repositioning and immobilizing the bone, if there is not a major displacement. In other cases, such as in fractures with multiple fragments, osteosynthesis surgery is indicated to stabilize the fractures, which allows proper alignment and alleviates the pain. Once the fractured bone has been successfully aligned, immobilized, and healed, the patient will typically undergo a period of rehabilitation to strengthen the injured area before total recovery.

In addition to the fracture injury itself, several associated clinical situations require further long-acting pain relief. First, some fractured bones are difficult to immobilize and cause severe pain, such as midshaft clavicle fractures, sternal fractures, and scapular fractures [2]. Indeed, even patients that do not meet indication criteria for surgery may still seek surgical intervention due to intolerable pain under conservative treatment [3,4]. Secondly, pain management allows patients to resume normal life activities earlier and accelerates rehabilitation during the treatment of some bone fractures, such as rib fractures, pubic fractures, and spine compression fractures [5,6,7,8,9]. Thirdly, for elderly or sick patients, the risks involved in surgical procedures are too high, and, thus, surgery is not an option, for example, to treat hip fractures [10]. This might have devastating outcomes in hip fracture patients, since the association of bed rest treatment with mortality is 2.5-fold more likely than that of patients treated operatively [11,12,13,14]. Nevertheless, pain relief is critical for the repair and rehabilitation of hip fractures, regardless of the initial treatment. Finally, pain management can improve patient’s performance during rehabilitation, range of motion of the joint, and life quality. This decreases complication rates, especially for knee and shoulder fractures, which are associated with local stiffness due to high postoperative pain [15,16]. Therefore, effective analgesia is an important component of patient care, which can improve clinical outcomes and patient satisfaction [1,17], not only for bone fracture treatment but also for other painful situations, such as surgical wound care [18].

**Figure 1:**
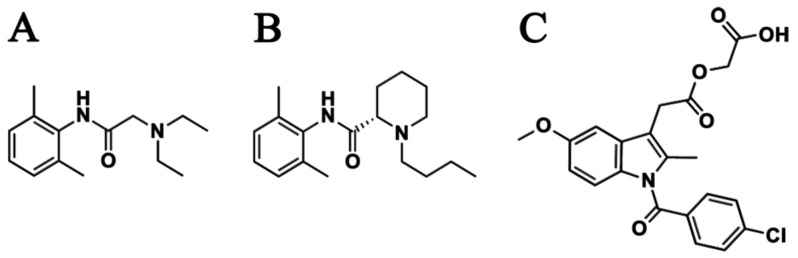
Chemical structures of (A) lidocaine, (B) levobupivacaine, and (C) acemetacin.

Methods for clinical pain control include: oral medications, such as opioids and non-steroid anti-inflammatory drugs (NSAIDs; e.g., naproxen, tiaprofenic acid, and acemetacin); local applications of analgesics; intravenous analgesia; or a combination of any of these. However, oral and intravenous administrations are often insufficient to effectively relieve severe acute pain in a specific area (e.g., bone fracture site), and the administration of high doses to compensate for this can lead to adverse effects. Consequently, the local delivery of adequate doses of analgesics to the target site represents a better strategy for pain relief. Among other analgesics, several studies indicate that lidocaine, bupivacaine, and NSAIDs infusions can effectively reduce pain [6,19,20,21]. Levobupivacaine is an amino-amide local anesthetic drug belonging to the family of n-alkylsubstituted pipecoloxylidide. This anesthetic is usually used for local anesthesia, including infiltration, nerve block, ophthalmic, epidural, and intrathecal anesthesia [3,12,19,20,21,22,23]. Acemetacin, on the other hand, is effective in the treatment of osteoarthritis, rheumatoid arthritis, ankylosing spondylitis, and other types of rheumatoid inflammation, as well as in post-operative and post-traumatic pain [21]. Finally, lidocaine is a local anesthetic and antiarrhythmic drug [6,19,20]. Figure 1 shows the chemical structures of lidocaine, levobupivacaine, and acemetacin.

Despite their effectiveness, locally applied anesthetics are expected to be completely depleted within two days after conventional administration. To overcome such fast metabolization rates, various delivery vehicles such as hydrogels [24,25] have been developed to provide spatial and temporal control over the release of various therapeutic agents, including small-molecule drugs, macromolecular drugs, and cells [26,27,28]. Owing to their tunable physical properties, controllable degradability, and capability to protect labile drugs from degradation, hydrogels serve as a platform in which various physiochemical interactions with the encapsulated drugs control their release. In addition, biodegradable polymers have also been widely used for biomedical applications. In addition to their known biocompatibility and biodegradability, these polymers are able to provide sustained and target-directed drug releases for several purposes, including cancer treatment, growth stimulation, and infection control [17,18,23,29]. Among various polymeric materials available for formulation of local release systems, poly(D,L-lactide-co-glycolide) (PLGA) is one of the most widely used because of its ability to transport drugs at high levels to target regions, where it provides a controlled released of bioactive molecules [30,31]. The material, approved by the US Food and Drug Administration, is also safe for use in clinical settings due to its low toxicity and minimum inflammatory effects. Polymers, such as PLGA, are used to produce micro- or nanoparticles containing bioactive agents using several techniques. Electrospraying is a robust and versatile technique for the manufacture of micro- and nanoparticulates of different materials. During the procedure, a metallic cone emits a steady microscopic liquid jet; under the action of an electrical potential, the jet breaks up periodically into uniformly sized droplets. The technique possesses enormous applications in the pharmaceutical and biomedical arenas [32,33]. 

Therefore, to assess whether an analgesic-containing microparticle system is able to effectively relieve bone fracture pain, we used electrospraying technology to develop biodegradable levobupivacaine/lidocaine/acemetacin-containing PLGA microcarriers. Previous studies reported that the use of multi-analgesics exhibit superior outcomes than the use of single drugs in pain relief [34,35,36]. While lidocaine and levobupivacaine are fast and short-term anesthesia, acemetacin belongs to long-duration NSAIDs. We hypothesized that the combined delivery of acemetacin, lidocaine, and levobupivacaine to the fracture site would result in a superior and sustained pain relief of bone fractures [3,17]. After electrospraying, the particle sizes were assessed by scanning electron microscopy (SEM). Meanwhile, the in vitro release patterns of analgesics from the microparticles were evaluated utilizing high-performance liquid chromatography (HPLC). Additionally, the in vivo analgesics release was investigated by injecting drug-loaded microparticles to bone fracture sites of rats. Finally, the efficacy of the microparticle system in rats was confirmed using animal activity cage experiments.

## 2. Results

### 2.1. Characterization of Electrosprayed Particles

**Figure 2:**
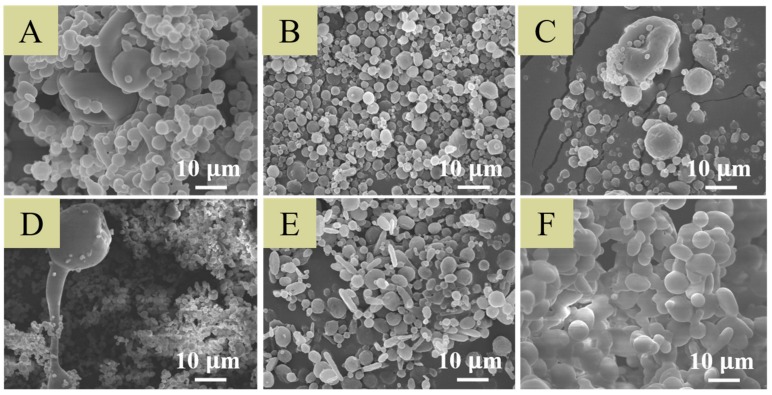
Field emission scanning electron microscopy images of analgesics-loaded microparticles subjected to various processing conditions after being gold-sputtered. Conditions were: (**A**) 9 kV, 1 mL/h, and 5 cm; (**B**) 9 kV, 1 mL/h, and 10 cm; (**C**) 9 kV, 1 mL/h, and 20 cm; (**D**) 6 kV, 1 mL/h, and 10 cm; (**E**) 15 kV, 1 mL/h, and 10 cm; and (**F**) 9 kV, 2 mL/h, and 10 cm for voltage, flow rate, and travel distance, respectively. Scale bar = 10 µm.

Figure 2 shows the SEM images of electrosprayed particles using varying preliminary electrospraying protocols. The analgesic-loaded microparticles prepared using condition B exhibited the most uniform size distribution (2.40 ± 0.82 µm). They were thus chosen for use in subsequent experiments.

**Figure 3:**
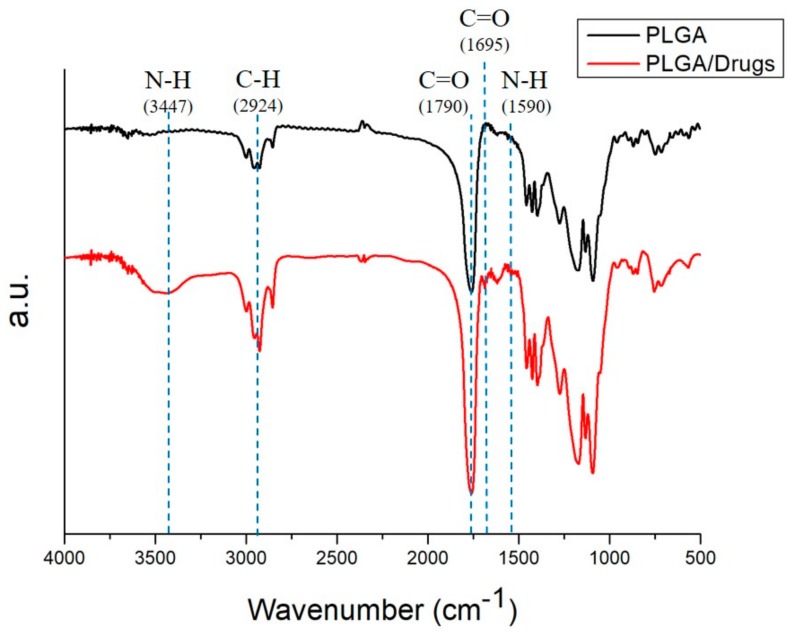
Fourier-transform infrared spectra of empty (black line) and analgesics-loaded (red line) electrosprayed microparticles.

Figure 3 shows the obtained Fourier-transform infrared (FTIR) spectra of pure and analgesic-embedded PLGA microparticles. The new absorption peaks at 3400 cm^−1^ and 1600 cm^−1^ might be attributable to the N-H bonds of levobupivacaine [37] and lidocaine [38]. The enhanced absorption at 1700 cm^−1^ corresponded to C=O bonds, primarily due to the addition of analgesics, including acemetacin [39]; and the absorbance peak near 2930 cm^−1^ may be the result of C-H bond enhancements in loaded drugs. Overall, the FTIR spectra demonstrated that the analgesics were successfully loaded into PLGA microparticles.

**Figure 4:**
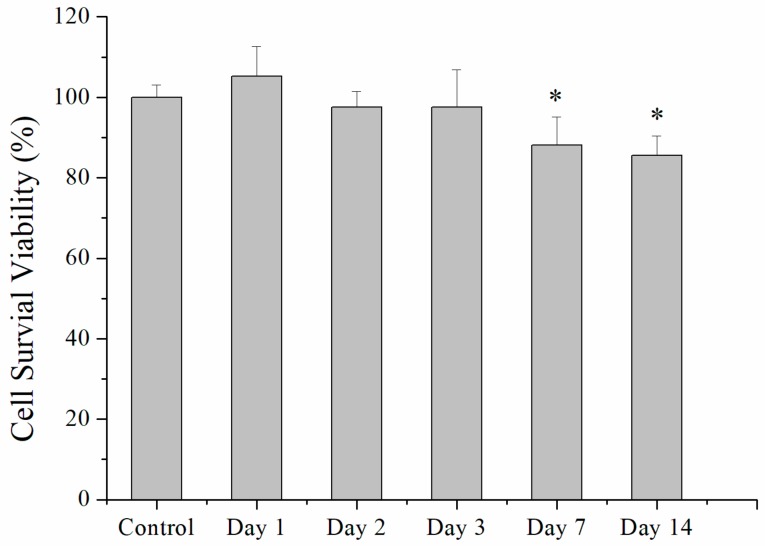
Cell viability of human fibroblasts treated with the eluents from analgesics-loaded microparticles incubated in phosphate-buffered saline for different periods. The asterisks denote significant differences from the control group. (*N* = 3, * *p* < 0.05).

The eluent from drug-loaded microspheres showed no signs of influencing the cell proliferations at days 1, 2, and 3, whereas fibroblast viability slightly decreased when treated with eluents from days 7 and 14 (Figure 4). The pH values of the eluents were 6.58 ± 0.11, 6.83 ± 0.07, 6.60 ± 0.10, 6.40 ± 0.36, and 6.28 ± 0.03 at 1, 2, 3, 7, and 14 days, respectively. Thus, the reduced viability of cells treated with eluents from days 7 and 14 may have been caused by this small decrease in pH as PLGA was degraded over time. This demonstrated that the PLGA microparticles are a potential good carrier for the delivery of analgesics.

### 2.2. In Vitro Release of Analgesic-Eluting Microparticles

**Figure 5:**
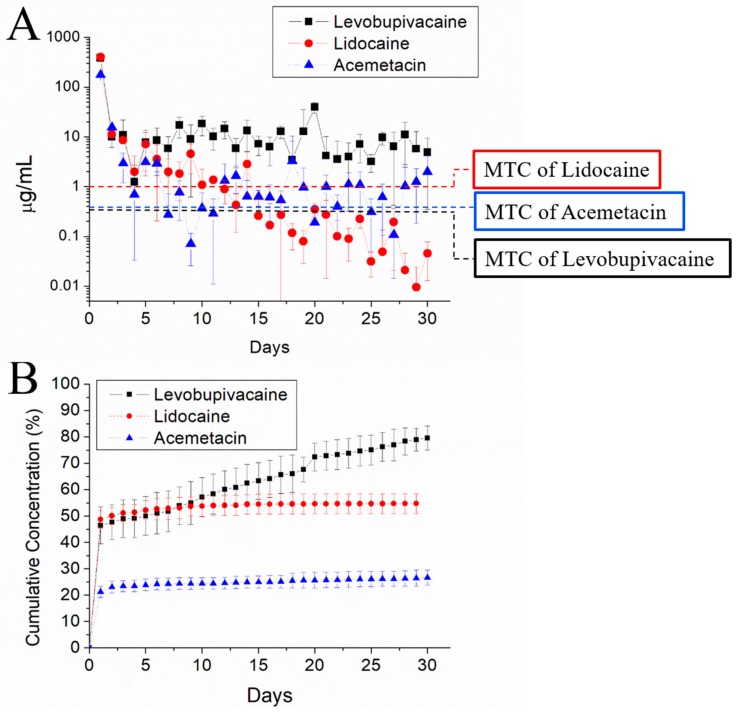
Release curves of analgesics from the electrosprayed microparticles during incubation in vitro. (**A**) Daily releases of levobupivacaine (black squares), lidocaine (red circles), and acemetacin (blue triangles). The dashed lines indicate the minimum therapeutic concentration (MTC) for each analgesic. (**B**) Cumulative values over the 30-day period.

Figure 5 illustrates the elution characteristics of levobupivacaine, acemetacin, and lidocaine from the electrosprayed analgesic-loaded microparticles in vitro. Biphasic release curves could be observed, characterized by a sharp rise at the first 2–3 days, followed by gradually decreasing release rates. Microparticles released high concentrations of levobupivacaine above the minimum therapeutic concentration (MTC) for more than 30 days. In the case of acemetacin and lidocaine, the released concentrations were kept above their MTC thresholds for 6 and 10 days, respectively.

### 2.3. In Vivo Release and Pain Relief

**Figure 6:**
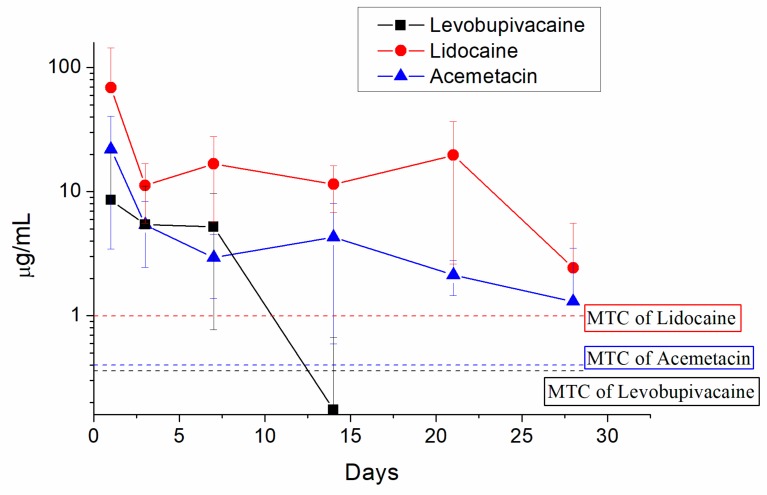
In vivo release curves of analgesics to the area surrounding the bone fracture site treated with analgesics-loaded microparticles. The levels of levobupivacaine (black squares), lidocaine (red circles), and acemetacin (blue triangles) were measured in the tissues 1, 3, 7, 14, 21, and 28 days after the treatment. The dashed lines indicate the minimum therapeutic concentrations (MTC) for each analgesic.

Figure 6 shows the measured drug concentrations in the tissues surrounding the fracture site. Analgesic-eluting microparticles offered long-term liberation of high levels of levobupivacaine, acemetacin, and lidocaine. The concentrations of acemetacin and lidocaine in the surrounding tissues were above the MTC levels for more than 28 days. For levobupivacaine, drug levels were maintained above its MTC only during the first seven days in the tissues around the fracture.

**Figure 7:**
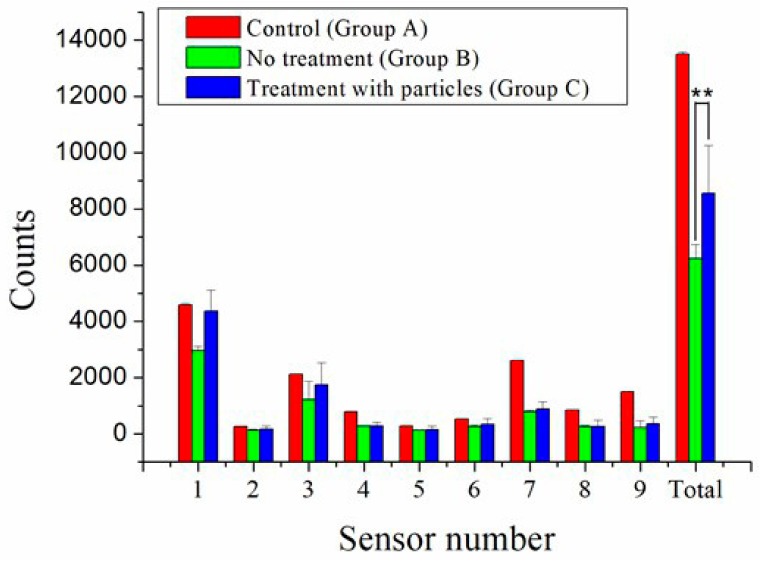
Rat locomotor activity recorded in the activity cages. Group A (red columns) was the control untreated group. Group B (green columns) was subjected to the experimental bone fracture procedure without any other treatment. Group C (blue columns) was subjected to the experimental bone fracture procedure followed by the administration of analgesics-loaded microparticles to the bone fracture site. The asterisks denote a significant difference (*p* < 0.01) between groups B and C.

The activity records of rats over the postoperative period of 7 days are shown in Figure 7. The greatest number of records at sensor number 1 was caused by the frequent visits around the area of the food and water supplies. The total triggered counts from all sensors were 13,492 ± 73, 6242 ± 504, and 8547 ± 1713 in groups A, B, and C, respectively. The activity of animals in control group A was significantly higher than those in groups B and C, highlighting the adverse effect of the surgical bone fracture on the animals’ activity. However, the activity index in group C was higher than that in group B, demonstrating that the animals that had been treated with the biodegradable analgesic-eluting microparticles showed greater activity levels.

## 3. Discussion

Some fractures, such as clavicle shaft fractures [40,41], rib fractures [17], scapular fractures [42,43], and fibular shaft fractures, can be treated without operation due to the high success rate of the conservative treatment. In these cases, the reduction in discomfort with a good pain control strategy becomes the main goal of patient care during fracture healing. Postoperative pain control is also important to allow a better recovery and reduce complications, for example, in intra-articular fractures such as tibial plateau fractures, proximal humeral fractures, and distal femur fractures. Proper pain control can not only keep patients comfortable, but it can also accelerate recovery and may reduce the risk of developing certain complications, such as pneumonia and blood clots. An ideal pain management plan should have a persistent effect and be easy to apply.

There are currently several kinds of treatments for fracture pain, such as oral medication, ice pack therapy, topical anesthetics, and injectable anesthetics. However, most of these do not relieve pain for more than one day, a short period that is far away from the need of serious injuries, such as bone fractures [19]. For example, intolerable pain may persist for as long as 1–2 months for a simple fracture without surgical fixation. In the case of a postoperative condition, the duration of moderate pain and inflammation is 2–4 weeks. Therefore, an ideal analgesia protocol should provide sustainable pain relief for at least four weeks. Moreover, in order to extend the action of the analgesics, a continuous intravenous infusion may be used. This kind of drug administration is usually provided by a continuous medicine pump, which requires specialized care, medication refilling, and hospitalization for side effect supervision.

On the other hand, some pain management drugs have been administered using a catheter into the epidural space around the spinal cord or nerve root, whereby the local anesthetics drugs can be continuously delivered to achieve a sustained analgesic effect. Nevertheless, this procedure requires highly specialized knowledge and possess several risks, such as catheter misplacement and nerve injury [3,22]. Invasive analgesia procedures are also associated with complications, such as infections, dural tears, and loss of motor function, which impairs post-injury rehabilitation. Still, oral medication is the most used method for pain control during conservative treatments of bone fractures, which may result in systemic side effects under long-term use, such as gastric ulcers, renal failure, and addiction. 

In this study, we successfully developed analgesic-loaded PLGA microspheres that provide sustained release of analgesics for pain relief post-surgery by employing the electrospraying technique [44]. Among various biomaterials, PLGAs have been one of the most researched biodegradable polymeric materials, mainly owing to their versatility, tailorable biodegradability, and excellent biocompatibility. Systems based on PLGA have been extensively investigated for their target specificity and controlled transport of micro/macromolecules [45]. The electrospraying, with charged droplets, possesses advantages over conventional mechanical spraying systems. In this process, the solution is charged by the nozzle, which is connected to a high-voltage power supply. The electrostatic force incurred by these charges surmounts the surface tension of the solution and splits the solution jet into very small droplets. Among various processing conditions, condition B (9 kV, 1 mL/h, and 10 cm) was found to provide the smallest and most uniform particles. The applied voltage is a required component in achieving sustainable solution-jet mode during electrospraying. However, if the applied voltage is too high, the polymeric solution may be overstretched, and particles with tails or irregular shapes could be seen. There is a positive correlation between the PLGA solution flow rate and particle size [44]. As the flow rate decreased, the size of the electrosprayed microparticles decreased accordingly. The smallest flow rate (1 mL/h) employed in the experiments thus produced particles with the smallest size distributions. On the other hand, during the electrospraying process, solvent evaporation and polymer chain diffusion occur during the travel of solution droplets to the collector. An appropriate travel distance is thus important to produce microparticles with desired sizes and morphology.

Generally, drug releases from pharmaceutical-embedded biodegradable particles includes three phases, including an initial burst release, a diffusion-dominated elution, and a degradation-dominated release. After electrospraying, while most of the molecules are encapsulated inside the microparticles, some of the pharmaceutical compounds may be located on the particles’ surfaces. This in turn results in an initial drug-release burst. After that, the drug-release profiles were governed by diffusion, as well as by degradation of the polymeric materials. Thus, a fairly constant decline in the release rate of the analgesics was observed. Overall, the electrosprayed microparticles could elute high levels (well over the minimum therapeutic concentrations) of lidocaine and acemetacin and levobupivacaine at the fracture site for more than 28 days and 12 days, respectively. Levobupivacaine exhibited much faster release rates than lidocaine. This might be due to the fact that the lipophilicity of levobupivacaine (Log P: 3.9) is greater than that of lidocaine (Log P: 2.26) [46]. The in vivo release of levobupivacaine was thus rapid, and the total release period decreased accordingly. Furthermore, the analgesics provided a persistent local pain control in rats without any motor dysfunction and systemic problems when being administered around the fracture site. This approach provides advantages in terms of long-term pain control for bone fractures.

As a drug delivery system, microparticles have been shown to improve the therapy efficiency [47], thus becoming more and more popular in research owing to the adequate preservation of drugs and biomolecules from degradation, as well as the ability to realize a controlled drug liberation at the target site. To adjust the delivery and liberation of drugs, it is important to controllably prepare polymeric microparticles with desired sizes and narrow size distributions. The analgesic-loaded microparticles developed in this study can be easily administered into the body by a direct injection via syringe due to their small sizes, as well as narrow size distributions (2.54 ± 1.08 µm). Therefore, they can be applied to a wide variety of pain control strategies and offer the advantages of pain relief, low cost, short hospitalization periods, better rehabilitation, and improved life quality. In addition, the experimental results demonstrated a significant increase of total activity counts (*p* < 0.01) one week post-surgery for the animals that gained the injection of analgesic-loaded microparticles when compared to those without the injection. This further testifies the efficacy of analgesics-loaded microparticles in the relief of facture pain.

Despite this study has obtained some experimental findings, few limitations exist. The first one is the rather small number of experimental animals enrolled in this work. The second limitation appears to be the insufficient period of the animal activity assessment. Third, the relationship between the findings in this work and the fracture pain control in humans remains uncertain and requires further investigation. Fourth, the experimental results suggest that the variation of pH values due to the degradation of PLGA may affect cell proliferation. This may need some additional explorations for future works. Furthermore, adequate drug concentrations and injection vehicles still need further examination.

## 4. Materials and Methods

### 4.1. Production of Biodegradable Analgesic-Eluting Microparticles

All materials used in this study, including PLGAs (50:50), levobupivacaine hydrochloride, acemetacin, lidocaine hydrochloride, and dichloromethane (DCM), were obtained from Sigma-Aldrich (St. Louis, MO, USA).

Analgesic-eluting microcarriers were produced utilizing a lab-made electrospraying device, which included a syringe with a 20-gauge needle (internal diameter of 0.60 mm), ground electrode, collector, and high voltage supply. Vehicle (420 mg PLGA) and active pharmaceutical ingredients (20 mg levobupivacaine hydrochloride, 20 mg acemetacin, and 20 mg lidocaine hydrochloride) were first dissolved in 6 mL of DCM and mixed using a magnetic stirrer at ambient temperature (25 °C) for 1 h. To determine the optimum electrospraying parameters, preliminary electrospraying protocols were conducted according to the conditions listed in Table 1. The solutions were delivered by a syringe pump with a volumetric flow rate of 1.0 or 2.0 mL/h. The needle was connected to a high voltage supply with a positive DC voltage of either 6, 9, or 15 kV. The distance between the needle tip and the ground electrode was set at 5, 10, or 20 cm.

### 4.2. Scanning Electron Microscopy

A field emission scanning electron microscope (SEM) (FESEM; JSM-7500F, Jeol, Tokyo, Japan) was employed to assess the diameters of microparticles after they were sputtered with gold. The size distribution of the electrosprayed particles was measured by an ELSZ-2000 particle size analyzer (Otsuka Electronics, Osaka, Japan).

### 4.3. Fourier-Transform Infrared Spectrometry

To evaluate the spectra of both pure PLGA and analgesic-incorporated microparticles, a Fourier-transform infrared spectrometry (FTIR) was used. The analysis was completed using a Nicolet iS5 spectrometer (Thermo Fisher Scientific, Waltham, MA, USA). The resolution was set at 4 cm^−1^ (32 scans). The particles were embedded into pressed KBr discs, while the spectra were monitored within the range of 400–4000 cm^−1^.

### 4.4. In Vitro Release of Analgesics

The release of analgesics from microparticles in vitro was characterized by an elution method. Microparticles of 2 mg were added to test tubes (*N* = 3) containing 1 mL phosphate-buffered saline (PBS) solution (0.15 mol/L, pH 7.4) at 37 °C in an isothermal oven. The solution was gathered and substituted with new PBS (1 mL) daily for 30 days. The levels of analgesics released into the PBS solutions were quantified by HPLC [18] using a L-2200 System (Hitachi, Tokyo, Japan). All experiments were performed in triplicate (*N* = 3).

### 4.5. Cell Culture

Cytotoxicity of electrosprayed drug-loaded PLGA microspheres was assessed using Cell Counting Kit-8 (CCK-8) assays (Sigma-Aldrich, St. Louis, MO, USA) of cell viability. Collected eluents at 1, 2, 3, 7, and 14 days were put onto 96-well culture plates. Commercially available human foreskin fibroblasts (3T3; Food Industry Research and Development Institute, Hsinchu, Taiwan) were seeded (1 × 10^4^ cells/well) in Dulbecco’s modified Eagle’s medium at 37 °C under the incubating condition that includes 5% CO_2_ and 95% ambient air for 48 hrs. Cell viability was recorded by CCK-8 assays and characterized using an ELISA reader (*N* = 3).

### 4.6. Rat Femur Shaft Fracture Model: Surgical Procedure and Animal Care

**Figure 8:**
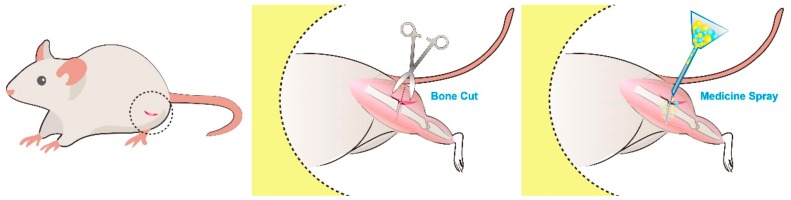
Femur shaft fracture surgical procedure. The lateral part of the middle-thigh region was disinfected, and a 2 cm skin incision was made (left panel). The femur shaft was exposed by blunt dissection of the surrounding musculature. The osteotomy was performed in a short oblique direction with a bone cut (middle panel). The analgesics-incorporated microparticles were directly sprayed on the fracture site (right panel).

All animal experimental procedures were approved by the Institutional Animal Care and Use Committee of Chang Gung University, and all studied animals were handled according to the regulations of the Ministry of Health and Welfare of Taiwan under the supervision of a licensed veterinarian (IACUC Approval No.: CGU106-092). Wistar rats weighting 200–300 g were selected for the in vivo experiments: 8 rats were used for the in vivo drug-release studies, while the other 15 rats were assigned to animal activity tests. Rats received general anesthesia by an inhalation of isoflurane (Aesica Queenborough, Queenborough, UK) in an anesthesia box (40 × 20 × 28 cm). Anesthesia was retained during the entire duration of the surgical procedures via mask isoflurane inhalation. A 2-cm-long skin incision was made at the lateral side of the right femur shaft, followed by the blunt dissection of the muscle until the exposure of the middle-third of the femur shaft. A simple femur shaft fracture was created, and then approximately 20 mg of analgesic-embedded microparticles were placed around the fracture. After the procedure, the fascia and skin were sutured with 3–0 Vicryl sutures (Johnson & Johnson, New Brunswick, NJ, USA) to finish the surgery (Figure 8).

For the drug-release experiments, 8 rats received general anesthesia by inhalation of isoflurane at 1, 3, 7, 14, 21, and 28 days post-operation. Soft tissues around the fracture were collected [18] (approximately 0.05 g each) by opening the wounds for drug level evaluations, and the wounds were then closed with 3–0 Vicryl sutures. The levels of analgesics in tissues were characterized by the HPLC assay.

### 4.7. Post-Surgery Activity Assessment

**Figure 9:**
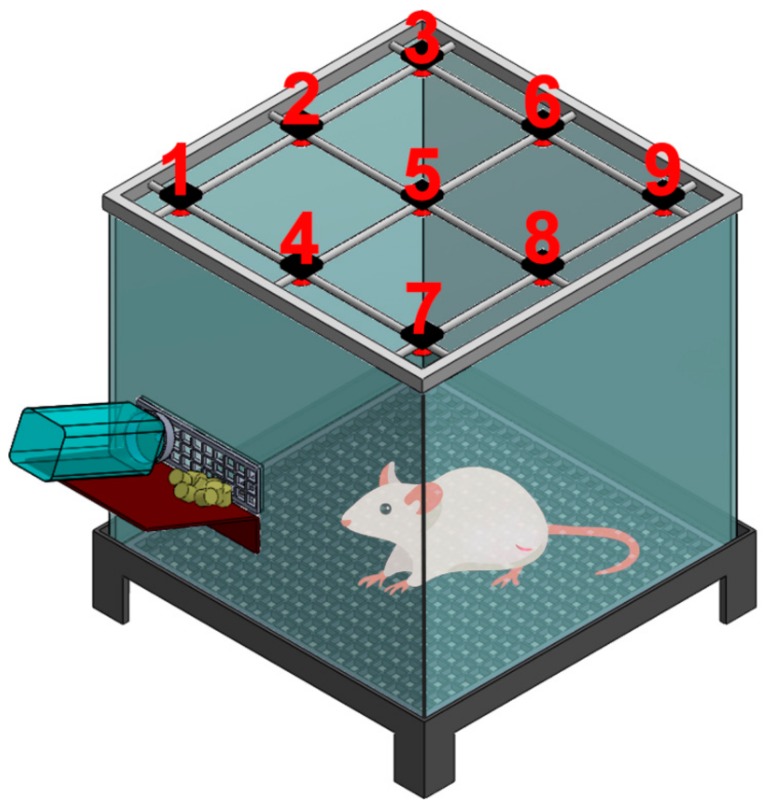
Schematic representation of the animal activity cage.

All experimental procedures gained approval from the Chang Gung University Institutional Animal Care and Use Committee (CGU13-039), and animal care adhered to the guidelines of the Department of Health and Welfare, Taiwan. The other 15 rats designated for animal activity tests were divided into three groups, with five rats in each group (*N* = 5). Rats in group A did not go through any procedure and served as a control group. Group B was subjected to the bone fracture surgery only (with no administration of microparticles), whereas group C was subjected to the surgery and received the administration of analgesic-loaded microparticles at the fracture site. After the operations, the activity of each animal in the different groups was assessed using an animal activity cage. Figure 9 shows schematically the cage, which has a dimension of 50 × 50 × 50 cm. On top of the cage, nine diffusion-scan-type photoelectric switch sensors that possess self-contained amplifiers (HP100-A1; Azbil Corp., Tokyo, Japan) were equipped. The sensors recorded the migrations of a rat in the cage. When the animal migrates from one region of the cage to another, the sensor in the “arriving” region will be triggered. A microprocessing unit furnished with an acquisition interface was used to monitor the total number of triggers. The activity of each animal was evaluated for seven days.

### 4.8. Statistics and Data Analysis

Data were analyzed by one-way analysis of variance (ANOVA) and *p*-values below 0.05 were considered to indicate statistically significant differences. The data were analyzed using SPSS software (version 17.0 for Windows; SPSS Inc, Chicago, Illinois, USA).

## 5. Conclusions

We have successfully developed biodegradable analgesic-loaded PLGA microparticles that provide the sustainable release of levobupivacaine, lidocaine, and acemetacin to the target site utilizing the electrospraying technique. Electrosprayed microparticles could elute high levels of lidocaine/acemetacin (well over the minimum therapeutic concentrations) and levobupivacaine at the fracture site for more than 28 days and 12 days, respectively. Rats treated with analgesic-eluting microparticles exhibited greater activity than those without treatment. Electrosprayed analgesics-loaded microparticles demonstrated their effectiveness and long-acting pain relief during healing of fracture injuries. Eventually such drug-eluting microparticles may be used for the pain control of various bone fractures in humans.

## Figures and Tables

**Table 1:** Parameters used in the preliminary electrospraying protocols for microparticles production.

Condition	Voltage (kV)	Flow rate (mL/hr)	Travel distance (cm)
A	9	1	5
B	9	1	10
C	9	1	20
D	6	1	10
E	15	1	10
F	9	2	10
